# Early childhood cognitive development is affected by interactions among illness, diet, enteropathogens and the home environment: findings from the MAL-ED birth cohort study

**DOI:** 10.1136/bmjgh-2018-000752

**Published:** 2018-07-23

**Authors:** MAL-ED Network Investigators, Angel Mendez Acosta

**Affiliations:** Department of Nutritional Sciences, The Pennsylvania State University, University Park, Pennsylvania, USA

**Keywords:** child health, hygiene, nutrition, anaemia, parasitology

## Abstract

**Background:**

Millions of children in low-income and middle-income countries (LMICs) are at risk of not reaching their full cognitive potential. Malnutrition and enteric infections in early life are implicated as risk factors; however, most studies on these risks and their associations with cognitive development have failed to adequately account for confounding factors or the accumulation of putative insults. Here, we examine the interaction between infections and illness on cognitive development in LMIC community settings.

**Methods:**

As part of the Etiology, Risk Factors, and Interactions of Enteric Infections and Malnutrition and the Consequences for Child Health and Development (MAL-ED) longitudinal birth cohort study, children from eight LMICs were followed from birth to 24 months to understand the influence of repeated enteric infections on child growth and development. Here, data from six sites were employed to evaluate associations between infection, illness, the home environment, micronutrient intake and status, maternal reasoning, and cognitive development at 24 months.

**Results:**

Higher rates of enteropathogen detection and days with illness were associated with lower haemoglobin concentrations, which in turn were associated with lower cognitive scores at 24 months. Children with lower environmental health/safety scores and lower intakes of vitamin B_6_ and folate had more enteropathogen detections and illness. Strength of associations varied by weight-for-age in the first 17 days of life; lower weight infants were more susceptible to the negative effects of enteropathogens and illness.

**Conclusions:**

Enteropathogens were negatively related to child cognitive development. However, other factors were more strongly associated with child cognition. Targeting of interventions to improve cognitive development should include a focus on reducing frequency of illness, improving the safety and healthfulness of the child’s environment, and improving dietary intake.

**Key questions**

**What is already known?**

- Child cognitive development is complex and has multiple influencing factors.
- Several studies have reported a negative association between early childhood diarrhoea and child cognitive development, but most have not examined how multiple factors collectively predict cognitive development.

**What are the new findings?**

- Enteropathogen detection in both diarrhoeal and non-diarrhoeal stools is negatively related to child cognitive development.
- The effects of enteropathogens on cognitive development are mediated by illness and haemoglobin concentrations.

**What do the new findings imply?**

- Targeting of interventions to improve cognitive development should include a focus on reducing illness, and improving the child’s play area and dietary intake.

## Introduction

Cognitive ability refers to the degree to which a person can think, reason, understand and remember information, solve problems, and learn.^1^ Childhood cognitive ability has been shown to predict important outcomes including academic achievement, occupational success and social adjustment.^2^ The first 2 years of life represent a critical window for cognitive development.^3^ It is during this time that the foundations of sensory and perceptual systems that underlie language and socioemotional behaviour are formed. Cognitive functioning in early life predicts later cognitive competence,^4^ with assessments at 24 months of age being related to IQ at 8–9 years of age.^5^ Therefore, factors that disrupt brain development during these crucial years may have lasting consequences for later brain functioning. Identifying and assessing these disruptions in early life are necessary for targeting interventions to those children who may benefit the most.

Over 200 million children worldwide are not developing to their full cognitive potential.^6^ This loss of human potential is associated with >20% deficit in adult income with broader implications for national development.^6^ Ensuring optimal cognitive development benefits the social and economic growth of countries and helps to break the intergenerational cycle of poverty.^7^ Infectious diseases, environmental exposures (eg, heavy metals, violence, lack of adequate cognitive stimulation) and nutrient deficiencies are all known risk factors for poor cognitive development^8^ and are prevalent among children under 5 years of age in lower income and middle-income countries (LMICs).^8^

The combination of enteric infections and diarrhoea contributes to (and can result from) malnutrition.^9^ The combined effects of marginal diets, unsanitary environments, and repeated and persistent enteric infections^10 11^ are thought to impair cognitive development. Yet most studies done to date have failed to adequately control for these multiple risk factors, making it difficult to ascertain the effects of enteric pathogens on cognitive development.

The Etiology, Risk Factors, and Interactions of Enteric Infections and Malnutrition and the Consequences for Child Health (MAL-ED) study is a multidisciplinary, prospective, community-based birth cohort study at eight sites in countries with historically high incidences of diarrhoeal disease and malnutrition.^12^ Here we evaluate whether enteropathogens in early life, detected even in the absence of diarrhoea, relate to cognitive development at 24 months of age. We hypothesised that enteropathogen detection in stools in early life would be negatively associated with child cognitive development. We further hypothesised that the strength of association between infection and cognition would vary by weight in the first 17 days of life as this is a common indicator of children predisposed to poor health outcomes and likely reflects endowment from the prenatal environment.^13^

## Methods

### Study design and population

Data were collected between November 2009 and February 2014 at eight sites in LMICs: Dhaka, Bangladesh (BGD), Naushahro Feroze, Pakistan (PKN), Vellore, India (INV), and Bhaktapur, Nepal (NEB) in Asia; Fortaleza, Brazil (BRF) and Loreto, Peru (PEL) in the Americas; and Venda, South Africa (SAV) and Haydom, Tanzania (TZH) in Africa. Each site was charged with enrolling (within the first 17 days of life) and following a cohort of 200 children until 24 months of age. Exclusion criteria included very low birth weight (<1500 g), very ill, non-singleton, mother <16 years of age and family not expecting to stay in the community at least 6 months. Caregivers provided written consent. Specifics on the study sites and populations are described elsewhere.^12^

#### Enrolment

At enrolment, infants were weighed and measured by trained anthropometrists following standard procedures. Because weight at birth was not available at all sites for all infants (it was measured in the first 17 days of life for all infants in the study), and because date of enrolment varied across infants and sites, weight was converted to weight-for-age z-score (WAZ) using the WHO 2006 growth standards^14^ and used in the analyses.

#### Morbidity surveillance

Data on daily illness and treatment were collected biweekly via maternal report and/or researcher measurement during visits to each household. Researchers assessed for presence of vomiting, fever, symptoms of diarrhoea and acute lower respiratory infection (ALRI).^15^ Diarrhoeal episodes were defined as consecutive days with diarrhoea (≥3 loose stools in 24 hours or presence of blood in stool) separated by at least 2 days with no diarrhoea.^16^ ALRI was defined using a standard definition of an observation of (1) cough or shortness of breath (on the day of the visit or the previous day) and (2) a rapid respiratory rate on the day of the visit (average of two measurements taken by the fieldworker). Rapid respiratory rate was defined according to the WHO guidelines (<60 days old: ≥60 breaths/min; 60–364 days: ≥50 breaths/min; and ≥365 days of age: ≥40 breaths/min). The cumulative number of days with any of the symptom listed was calculated for each child and used in the analyses.

#### Enteropathogen detection and gut function

To identify enteropathogen exposure, research staff collected stool samples during each diarrhoeal episode, as well as non-diarrhoeal stool samples on a monthly basis. Both non-diarrhoeal and diarrhoeal samples were tested using conventional assays for an expansive panel of bacterial, viral and parasitic enteropathogens, with non-diarrhoeal stools tested monthly during the first year and quarterly thereafter for reasons of cost.^17^ The mean number of different pathogens detected per stool was calculated for each child using all protocol-compliant (collected on schedule and analysed for all pathogens) stools collected between 1 and 24 months of life. In these analyses, diarrhoeal and non-diarrhoeal stools were considered separately. Faecal and urinary biomarkers of gut inflammation and disruption were evaluated as follows: permeability and malabsorption (lactulose-mannitol test at 3, 6, 9 and 15 months); intestinal inflammation (faecal myeloperoxidase and neopterin); and protein-losing enteropathy (α-1-antitrypsin) detected in non-diarrhoeal stools at monthly intervals during the first year and quarterly during the second year of life.^18^

#### Diet and nutrient status

During the biweekly home visits, caregivers were queried about breast feeding and the introduction of non-breast milk foods. Intakes of complementary foods (ie, non-breast milk foods) were quantified monthly using the 24-hour recall method from 9 to 24 months, with nutrient intakes from preparations calculated from caregiver-reported recipes.^19^ Of particular interest were B vitamins (vitamins B_6_, B_12_ and folate) that have a known association with cognitive development,^20–23^ and protein intake from meat, fish and poultry (MFP protein) that are good sources of iron and zinc. The mean intakes for each micronutrient and macronutrient were calculated from the 16 monthly recalls for each child. Micronutrient status was assessed via blood and urine collection at 7 (6 for urinary iodine), 15 and 24 months of age, and included measures of iron (haemoglobin, plasma ferritin, soluble transferrin receptor), zinc (plasma zinc), vitamin A (plasma retinol) and iodine status (urinary iodine concentration).^19^

Alpha-1-acid glycoprotein (AGP) was measured to quantify systemic inflammation.

#### Socioeconomic status

Families were asked about household assets and income, type of sanitation, source of drinking water and handwashing behaviours, as well as maternal education at 6-month intervals. A socioeconomic index, the Water, Assets, Maternal education and household Income, was constructed from the socioeconomic status (SES) variables,^24^ and a crowding index (calculated as the mean number of people per room) was also used in the analyses.

#### Home environment

The Home Observation for Measurement of the Environment (HOME) inventory^25^ (infant/toddler version, Black *et al* modification^26^) was completed at 6 and 24 months of age to examine dimensions of the home environment known to be beneficial for child development. The HOME used in MAL-ED had 43 binary items that, during psychometric analyses,^27^ loaded on to three factors, one of which was retained in these analyses, namely the environmental safety and healthfulness around the child. This factor included questions on whether a stove was present in the play area, whether the play area was free from hazards and whether the house was relatively light.

#### Child development

The Bayley Scales of Infant and Toddler Development-III (BSID-III)^28^ was used to assess child development at 6, 15 and 24 months of age. This analysis focused on the 24-month cognitive scores as we sought to examine the association between enteric infections early in life and subsequent child development. Trained personnel with a background in psychology or child development administered the tests in a quiet, well-lit central location at each site on days when children were not ill. Approximately 6% of administrations were video-recorded and reviewed for quality control purposes.^29^ These quality control and assurance reviews revealed that the administration of the BSID-III in TZH deviated significantly from our protocol, which necessitated exclusion from analyses. Psychometric analyses (exploratory and confirmatory factor analyses) of the BSID-III scores were conducted to examine invariance of scores across sites (LL Pendergast *et al*, under review, invited resubmission) to determine whether the sets of questions were addressing the same underlying theoretical constructs in each site. Based on those analyses, 15 items were retained that reflected a variety of skills, including representational play, object assembly, matching pictures, attention to a story, and working with tools, puzzles and toys. The psychometric analyses also suggested that the questions performed differently in the NEB population compared with the other six (LL Pendergast *et al*, under review, invited resubmission). Consequently, we excluded the BSID-III data from NEB for the present analyses. Thus, our results are based on data from six sites and focus on the cognitive development outcome of the BSID-III. We have chosen not to report raw scores of cognitive development by site because of historical abuse and misinterpretation.^30–33^

#### Maternal factors related to child development

Given the known relation between child development and maternal reasoning ability, as well as maternal depressive symptoms, data were collected on these variables.^34 35^ Maternal reasoning ability was measured once with the Raven’s Progressive Matrices^36^ when the child was between 6 and 8 months of age. Psychometric analyses supported a single-factor score that was valid across all sites. Maternal depressive symptoms were measured when the child was 1, 6, 15 and 24 months using the Self Reporting Questionnaire,^37^ which contains 20 binary items. Psychometric analyses revealed a 16-item, one-factor structure with items reflecting internalising symptoms. This one-factor model was a good fit for the data at all sites, except BRF where validity was not supported statistically.^38^

### Statistical analysis

Based on extant literature and expert opinion, a theoretical framework was developed (online supplementary figure 1). Given the complexity of child cognitive development, we sought to develop a model which captured data in multiple domains that have been shown to influence child development. These domains include infection, illness, gut function, dietary intake and status, SES, psychosocial stimulation, size at birth, and maternal factors known to be related to cognitive development (such as maternal reasoning ability) (see online supplementary table 1). Our conceptual model was used to build a structural equation model (SEM). The advantage of SEMs is that they can explicitly capture the complicated pathways by which important variables are related. The breadth of data collected within MAL-ED was extensive and many variables addressed similar conceptual domains. As such, the selection of variables was based on (1) strong theoretical basis for association with cognitive development; (2) zero-order correlation with the BSID-III outcome variable; (3) non-redundancy with variables already included in the model (when redundancy within a domain occurred, we selected the variable that had the greatest association with the BSID-III); (4) evidence that the variable was measured well (eg, quality assurance, reliability, validity); and (5) low rates of missing data.

10.1136/bmjgh-2018-000752.supp1 Supplementary data

**Figure 1:**
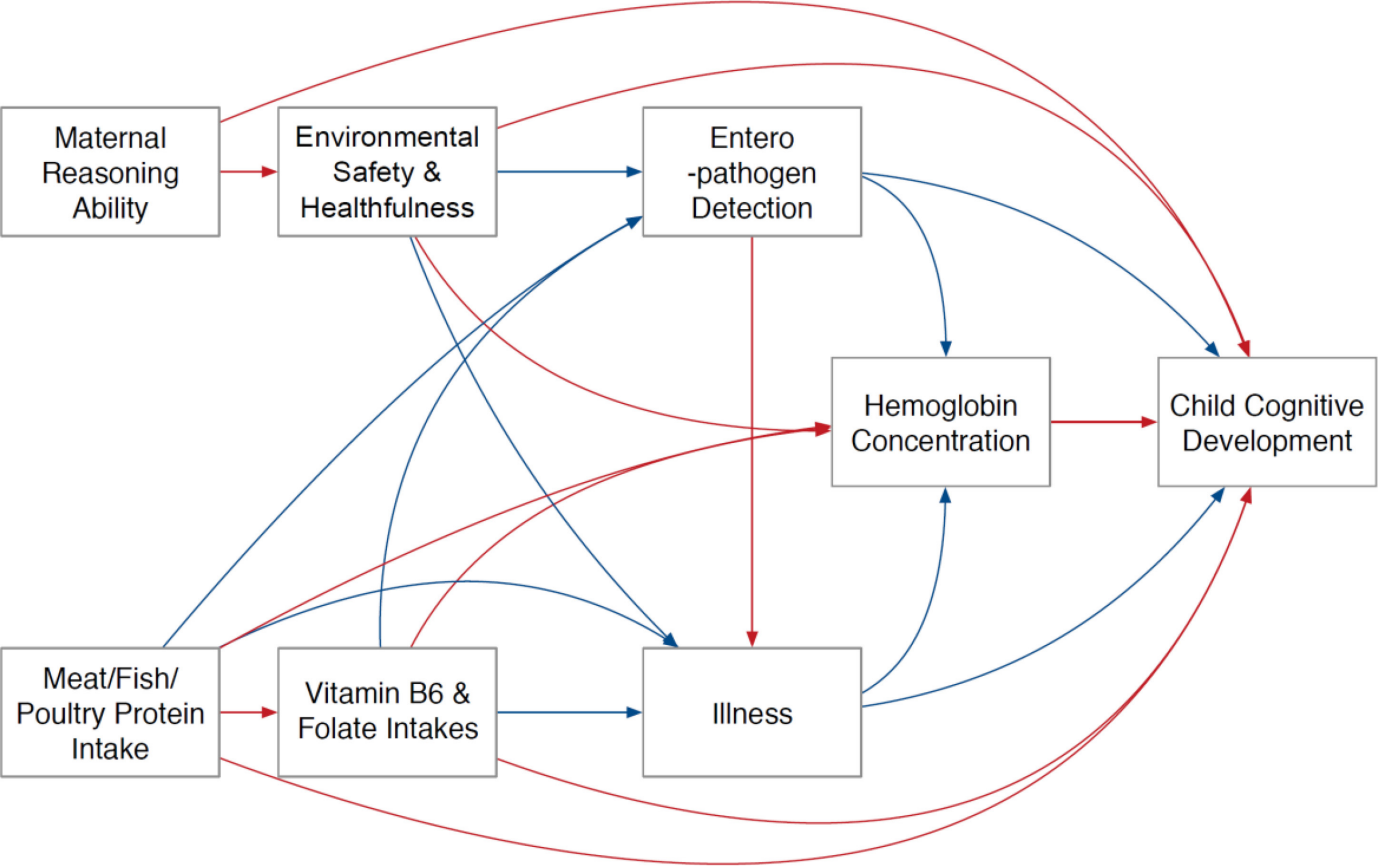
Path analysis model tested with hypothesised direct relationships between variables (blue arrows indicate negative associations and red arrows indicate positive associations).

Most of the domains that were hypothesised to be related to child development were represented in our final model. These included SES, dietary intake and status, enteropathogen infection, illness, and maternal factors. We considered size at birth by stratifying the data via weight in the first 17 days of life and running the models based on weight (see below). Of the domains, the following variables were selected in the final model (figure 1): mean home environmental safety and healthfulness factor score (6 and 24 months); mean (0–24 months) enteropathogen detection in non-diarrhoeal and in diarrhoeal stools (because inclusion of both stool types in the same model resulted in multicollinearity, therefore each was evaluated in a separate model); proportion of days with illness (fever, vomiting, diarrhoea or ALRI from 0 to 24 months); mean haemoglobin concentration (7, 15 and 24 months); mean intake of vitamin B_6_ and mean intake of folate from complementary foods (9–24 months); mean protein intake from meat/fish/poultry (9–24 months); and maternal reasoning ability score. Definitions for variables considered for inclusion and reasons for exclusion are outlined in online supplementary table 1.

As noted earlier, we hypothesised that the direct and indirect effects of repeated exposure to enteropathogens (in either diarrhoeal or non-diarrhoeal stools) on cognitive development would be different in children potentially predisposed to poor health outcomes. Although we did not collect any data prenatally, the child’s weight in the first 17 days of life is likely a reflection of the in utero environment. Therefore, we chose to stratify each model based on WAZ. The top third of observed WAZ was classified as higher weight (likely a reflection of a better in utero environment), and the bottom third classified as lower weight (likely a reflection of a less optimal in utero environment) (stratifying by thirds allowed for a large enough sample size in each tertile for comparison in our model). In our analyses, we systematically compared the fit among three models: (1) full (including all children), (2) lower weight children and (3) higher weight children. These models were run first considering enteropathogen detection rates in non-diarrhoeal stools, and then again using enteropathogen detection rates in diarrhoeal stools.

Multigroup path analysis was used to examine model equivalence, that is, that the same model fit equally for all groups (sites or weight classes), and fit was determined with a panel of goodness-of-fit metrics. Models were fit with robust maximum likelihood estimation (Mplus V.6.0) with bootstrapping to estimate indirect effects.

## Results

The characteristics of the variables included in the models can be found in table 1. Overall, 2145 children were enrolled in MAL-ED, with 1643 enrolled from the six sites included here (BGD, BRF, INV, PKN, PEL and SAV). Of these, 1298 were followed up to 24 months and 1169 (90%) underwent cognitive testing and are included here. In terms of specific sites, the percentage followed to 24 months of age ranged from 65% in PEL to 91% in PKN and INV. Of those followed to 24 months of age, those who underwent BSID-III testing ranged from 78% in BRF to 97% in PKN and INV. Approximately half of the sample were male and almost half were anaemic. Pooled across sites, the mean enteropathogen detections per stool were similar in diarrhoeal and non-diarrhoeal stools. There was substantial site-to-site variability in complementary diet (eg, a 35-fold difference in average MFP protein intake (0.27–9.59 g/day)) and in the enrolment WAZ (the range of site means was −1.39 to 0.12).

**Table 1:** Mean±SD for all sites combined and range of site-specific means for descriptive variables included in the model

Domain	Variable name	Overall Mean±SD	Range of site-specific means
Socioeconomic status	Environmental safety and healthfulness	3.32±0.88	2.16–3.87
Complementary food intake	Meat, fish and poultry protein intake (g/day)	3.96±4.56	0.27–9.59
	Vitamin B_6_ intake (mg/day)	0.66±0.6	0.26–1.57
	Folate intake (μg/day)	109±88	36.61–269.49
Micronutrient status	Haemoglobin concentration (g/dL)	10.91±1.17	9.79–11.57
Infection	Enteropathogen detection rate—non-diarrhoeal	1.12±0.48	0.65–1.63
	Enteropathogen detection rate—diarrhoeal	1.09±0.83	0.39–1.55
Illness	Fever	5.59±6.08	0.48–13.17
	Vomiting	4.11±9.21	0.18–10.73
	Diarrhoeal symptoms	3.02±4.72	0.37–9.11
	Acute lower respiratory infection	0.86±1.71	0.05–3.08
Maternal factors	Reasoning ability score	25.50±12.34	15.49–33.93
Anthropometry	Weight-for-age z-score at enrolment	−0.92±1.10	−1.39 to 0.12
Child development	24-month cognitive development score	6.85±4.26	2.41–9.84

### Model fit

Whether including enteropathogen detection from diarrhoeal or non-diarrhoeal stools, model fit varied based on the sample (full sample, lower weight or higher weight) (table 2). Models with the full sample had good fit^39^ (comparative fit index: CFI=0.99; root mean square error of approximation: RMSEA=0.066). Among lower weight infants the models had an excellent fit, but the model fit was relatively poorer among higher weight infants (RMSEA≥0.095). Statistically significant differences were found in the fit of the models of lower and higher weight infants (p<0.001; ΔCFI>0.01; ΔRMSEA>0.01), indicating that a single model structure did not describe the same relationships between variables based on weight at enrolment.

**Table 2:** Model fit indices from multigroup path analyses for full model, lower weight in the first 17 days of life and higher weight in the first 17 days of life of infants by type of stool examined

Model	Model fit indices			
	χ^2^	CFI	RMSEA	SRMR
Non-diarrhoeal stools				
Full model	18.43	0.990	0.066	0.015
Lower weight	0.35	1.000	0.000	0.003
Higher weight	13.91	0.969	0.095	0.023
Diarrhoeal stools				
Full model	26.38	0.984	0.082	0.020
Lower weight	0.76	1.000	0.000	0.005
Higher weight	18.14	0.958	0.111	0.029

CFI, comparative fit index; RMSEA, root mean square error of approximation; SRMR, standardised root mean square residual.

### Direct effects

**Figure 2:**
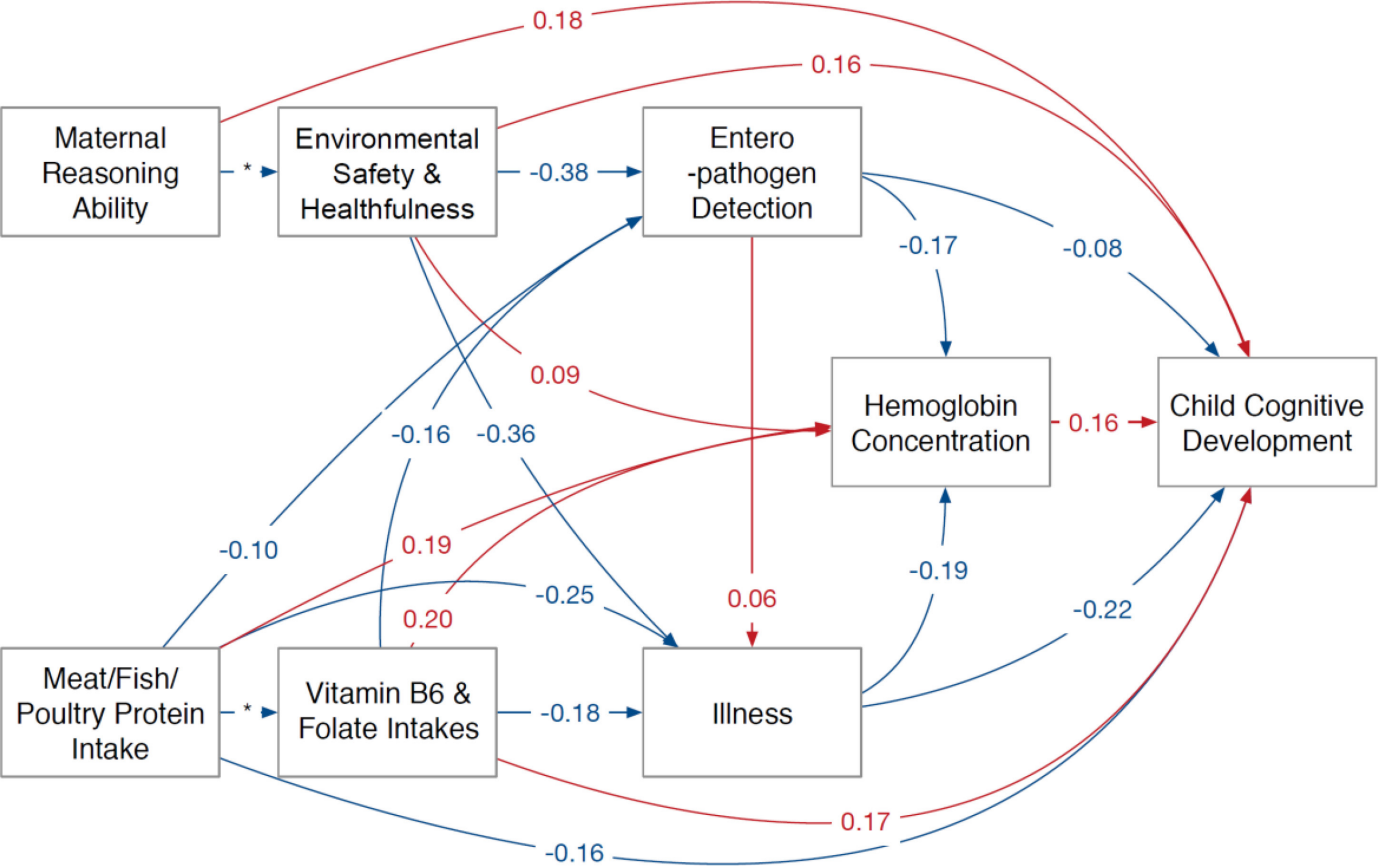
Standardised parameter estimates for direct mean effects from tested models. *Parameter fixed to allow for model estimation (blue arrows indicate negative associations and red arrows indicate positive associations).

**Figure 3:**
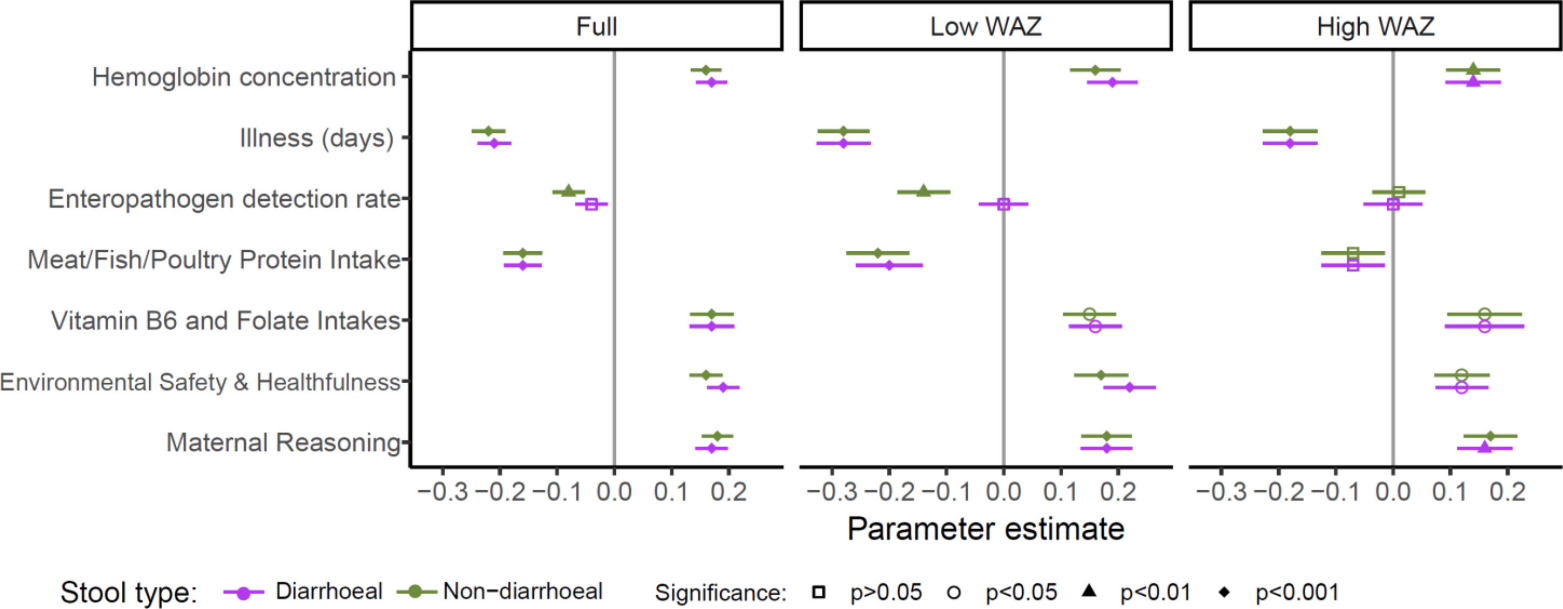
Mean direct effects of variables on child BSID-III cognitive score at 24 months, comparing models with enteropathogens from non-diarrhoeal (green) or from diarrhoeal (pink) stool samples and including all observations (full) or the lower and upper tertiles of WAZ at enrolment. The significance in the figure indicates whether or not the specific variable was significant in the path model, with the horizontal axis (parameter estimate) providing an indication of the strength of association. BSID-III, Bayley Scales of Infant and Toddler Development-III; WAZ, weight-for-age z-score.

Parameter estimates using enteropathogen detections from non-diarrhoeal stools and the full sample (ie, not stratified by WAZ) are shown in figure 2 (all were statistically significant at p<0.05). Enteropathogen detection rates, the proportion of days with illness and MFP protein intake from complementary foods were negatively related to cognitive development. Maternal reasoning ability, environmental safety and healthfulness, vitamin B_6_ and folate intakes, and haemoglobin concentration were positively related to cognitive development. The parameter estimates were similar whether the model included the pathogen detection rate in diarrhoeal or non-diarrhoeal stools (figure 3). Similarly, the mean parameter estimates were similar for the full model or comparing the lower and upper tertiles of WAZ at enrolment. Pathogens detected in diarrhoeal stools did not have a direct significant association with cognitive scores for any of the models tested (full sample, lower weight, higher weight). However, pathogens detected in non-diarrhoeal stools had a direct significant association with cognitive scores for the full sample and the lower weight models. Additionally, the association between environmental safety and healthfulness and cognitive scores was stronger in the lower WAZ children compared with higher WAZ children (parameter estimate of 0.17 compared with 0.12 in lower and higher enrolment WAZ, respectively; figure 3).

Better environmental safety and healthfulness scores, higher vitamin B_6_, folate and MFP protein intakes were associated with lower pathogen detection rates and the number of days of illness (figure 2). A higher rate of enteropathogen detections was weakly associated with an increase in the days of illness. The mean pathogen detection rate and illness were inversely related to haemoglobin concentration, whereas MFP protein intake, vitamin B_6_ and folate intakes, and environmental safety and healthfulness score positively related to average haemoglobin concentrations. These relationships were consistent for the WAZ tertiles, although the associations were more pronounced among lower weight infants than their higher weight counterparts.

### Indirect effects

Differences between lower and higher weight infants were also seen in the indirect effects in the model containing non-diarrhoeal stools (table 3). For lower weight infants, pathogen detection rate had the most notable indirect influence on cognitive development (via both illness and haemoglobin), whereas for higher weight infants, B vitamin intakes had the greatest indirect effect (via illness and pathogen detection). When examining the indirect effects in diarrhoeal stools (table 3), pathogen detection rates were significantly indirectly related to cognitive development in both lower and higher weight infants (via both illness and mean haemoglobin), with the relation being stronger in lower weight infants. As with the non-diarrhoeal stools, B vitamin intakes were more strongly related to cognitive development (via illness) in the higher weight infants.

**Table 3:** Standardised parameter estimates for indirect effects in tested models†‡

Predictor		Mediators	Outcome	Total indirect
Non-diarrhoeal stools				
Full sample	Illness	Haemoglobin	Cognitive score	−0.03***
	Non-diarrhoeal pathogen detection	Haemoglobin, illness	Cognitive score	−0.04***
	B vitamin intake§	Non-diarrhoeal pathogen detection, illness	Cognitive score	0.05***
Lower weight	Illness	Haemoglobin	Cognitive score	−0.04***
	Non-diarrhoeal pathogen detection	Haemoglobin, illness	Cognitive score	−0.05***
	B vitamin intake§	Non-diarrhoeal pathogen detection, illness	Cognitive score	0.04***
Higher weight	Illness	Haemoglobin	Cognitive score	−0.04*
	Non-diarrhoeal pathogen detection	–	Cognitive score	−0.01
	B vitamin intake§	Non-diarrhoeal pathogen detection, illness	Cognitive score	0.05**
Diarrhoeal stools				
Full sample	Illness	Haemoglobin	Cognitive score	−0.03***
	Diarrhoeal pathogen detection	Haemoglobin, illness	Cognitive score	−0.04***
	B vitamin intake§	Diarrhoeal pathogen detection, illness	Cognitive score	0.04***
Lower weight	Illness	Haemoglobin	Cognitive score	−0.04**
	Diarrhoeal pathogen detection	Haemoglobin, illness	Cognitive score	−0.06***
	B vitamin intake§	–	Cognitive score	0.02
Higher weight	Illness	Haemoglobin	Cognitive score	−0.04*
	Diarrhoeal pathogen detection	Haemoglobin, illness	Cognitive score	−0.04**
	B vitamin intake§	Diarrhoeal pathogen detection, illness	Cognitive score	0.05*

***P<0.001, **p<0.01, *p<0.05. Statistically significant relationships are in bold.

†Parameter estimates (ie, relationships between variables) are often much smaller in structural equation model analyses than in linear regression analyses because so many variables are accounted for. However, small relationships can be meaningful, especially when multiple paths of influence are involved.

‡Number of children included in model by site:

Full sample model: BGD=184; BRF=128; INV=227; PKN=197; PEL=242; SAV=195.

Lower and higher weight models: BGD=61; BRF=43; INV=76; PKN=66; PEL=81; SAV=65.

§Comprised the average vitamin B_6_ and the average folate intakes (as two separate variables) from 9 to 24 months.

BGD, Bangladesh; BRF, Brazil; INV, India; PEL, Peru; PKN, Pakistan; SAV, South Africa.

### Influence of demographic variables—sex and site

The patterns of relationships between variables were similar at all sites. Comparison of the model goodness of fit when additional stratification by sex or site was included suggested that the models met the criteria for partial configural invariance across sex and had an adequate fit at each of the six sites (online supplementary table 2).

## Discussion

Our findings indicate that children with poorer environmental safety and healthfulness scores and poorer intakes of vitamin B_6_ and folate had higher enteropathogen detection rates and greater numbers of days of illness. In turn, children who were ill more frequently had lower cognitive development scores, with evidence that a portion of this relationship was accounted for by a lower haemoglobin concentration. Importantly, we found that being at lower weight in the first 2 weeks of life exacerbated the negative relations between these and other variables in critical pathways related to cognition. This finding is consistent with results from the same study population that infants with low weight in the first 17 days of life were more likely to be stunted at 24 months of age.^13^

Several studies have documented a relation between early life diarrhoeal illness and poorer cognitive functioning,^40–42^ but most failed to account for potentially important confounding factors and have not elucidated potential pathways for the association between early life diarrhoeal illness and cognitive impairment. Fischer Walker *et al* 43 indicated the need for studies which examine the complex pathways by which infection, nutrition and the home environment impact child development, and which also account for mediating effects. Moreover, because most of the focus in this area of research has been on diarrhoea, our understanding of the relation between subclinical enteropathogens in particular and cognition is limited. It is against this backdrop that we prospectively examined associations between multiple variables and cognitive development using a model accounting for possible mediating and moderating effects. Ours is the first study to reveal that enteropathogen detection in non-diarrhoeal samples was negatively associated with cognitive development and that lower weight infants were more vulnerable to this effect than higher weight infants.

Pathogen detection was negatively related to cognitive development, but a stronger association was found between illness history and cognitive development. This was consistent whether pathogens were detected in diarrhoeal or non-diarrhoeal stools and independent of the weight class of infants in the first 17 days of life. Illness in general could alter mother–child interaction with subsequent repercussions for cognitive development. Respiratory illness could influence oxygen delivery with detrimental consequences for cognition, as could lower haemoglobin concentrations, which we found to be negatively associated with illness in all models (ie, more days of illness was associated with a lower haemoglobin concentration). Lower haemoglobin concentration has been related in other studies to lower quality mother–infant transactions and compromised brain development,^44 45^ and iron deficiency anaemia, specifically, is known to alter cognitive development.^46^ Respiratory illnesses that are not severe enough to cause an effect on oxygen delivery have also been shown to be related to brain development, specifically to learning impairment through deficient induction of long-term potentiation in the hippocampus. Therefore, ALRI may be related to cognitive development through a pathway that does not necessarily involve lower brain oxygen levels.

Additionally, we found that higher intakes of vitamin B_6_ and folate were associated with higher cognitive scores. Deficiencies of B vitamins, especially folate, vitamin B_6_ and vitamin B_12_, have been previously shown to relate to cognition.^47^ It is important to note that vitamin B_12_ intakes were strongly correlated with intakes of vitamin B_6_ in our sample but, given our variable selection process, only B_6_ was retained in the final model (as it had a stronger association with the outcome variable; as such, the findings reported here should not rule out a role for vitamin B_12_ intake). With respect to other nutrients, iron intake was not included in the model because we expected ferritin, a sensitive biomarker of iron status, to be more closely related to cognitive development. However, ferritin concentrations had small associations with cognition in our population compared with other variables; there was also redundancy in the model between the ferritin concentrations and other variables, and therefore ferritin was not retained in the final model. The inverse association between protein intake from meat/fish/poultry sources and cognition was unexpected and varied by site. The relationships are complex however, as greater intakes of protein from meat/fish/poultry were also related to higher mean haemoglobin concentration, to less time spent with illness and with lower rates of enteropathogen exposure—all associated positively with cognition. Variation in intake from complementary foods is reflective of variation in the duration of breast feeding,^48^ and thus lower intakes may reflect differences in breastfeeding practices. We note that approximately 80%–90% of children in the South Africa, Peru and Brazil sites were consuming flesh foods at 24 months of age, whereas consumption of flesh foods ranged from 30% to 60% in the remaining sites. Previous published work may shed some light on this unexpected finding,^49^ and we continue to explore this in order to publish an indepth analysis in a future manuscript. Contrary to our expectations, we did not find associations between breast feeding and child cognitive development. This may be because nearly all of the children were breast fed^48^ or an association between breast feeding and development may exist, but may not be evident until children are older. Although previous studies have reported an association between breast feeding and cognition,^50^ findings have been mixed and no previous studies have tested the complex model presented here. The lack of association between our measure of systemic inflammation (AGP) and cognitive scores was also an unexpected finding. We did not originally measure AGP with the intent of associating it with our cognitive outcomes but, rather, in order to adjust the micronutrient values as they are affected by inflammation. Nevertheless, if enteric infections disrupt the gut barrier, in turn causing systemic inflammation that alters the gut–liver–brain axis, we would have expected a relation between AGP and cognitive scores. One possibility for the lack of association is the fact that, as an acute phase protein, AGP can be elevated for many reasons and may not necessarily be reflective of events going on in the gut at that point in time.

In addition to the large number of enteropathogens assayed in both non-diarrhoeal and diarrhoeal stool samples, a particular strength of our study is the rigorous psychometric analyses to establish the appropriateness and robustness of analysing these data in a cross-site manner. Most studies overlook this crucial step when analysing data from psychological measures, and it hinders the interpretation and generalisability of their findings. Additionally, we used a harmonised protocol whereby fieldworkers at all sites were taught to collect data in the same fashion, and we had a comprehensive set of quality control measures to ensure that data included in our final analyses met the criteria of the harmonised protocol. Finally, we conducted detailed assessments of breastfeeding practices, intake of complementary foods and multiple biomarkers of nutrient status. The rigour of data collection did, however, mean that observations from some sites were excluded due to inconsistencies in administration or variance identified through the psychometric analyses. This analysis included children from six communities, but to understand mechanisms it would be necessary to have a much larger sample size than was possible here. The breadth of data collection meant that many variables from similar domains but with subtly different interpretations could not be analysed in the same model. Additionally, observations on some variables were missing, resulting in inability to include these variables in analyses due to the severe impact on sample size.

In these populations where subclinical enteric infection is common,^51^ our findings of negative consequences of enteropathogens in non-diarrhoeal stools on cognitive development especially for children born at a lower weight suggest that the global burden of disease and disability from enteric infections may be significantly underestimated if the focus is only on symptomatic diarrhoea. This final point resonates with our findings from analyses focused on growth outcomes.^48^ While interventions targeted at reducing enteropathogen exposure are necessary, results of our model indicate that other variables, in particular the frequency of illness and the safety/healthfulness of the home environment (which is strongly related to pathogen exposure), are more strongly associated with cognitive development than enteropathogen exposure directly. In addition, intakes of vitamin B_6_ and folate from complementary foods also play a positive role. It is important to remember that we examined the burden of various factors over time, and the life experiences captured in these children are commonly seen in LMICs. Results from this study, the most comprehensive of its kind to date across six diverse LMICs, indicate a need for holistic interventions targeting the safety/hygiene of the child’s play area, the treatment and prevention of a broad range of common childhood illnesses, and improvement of dietary intakes, specifically vitamin B_6_ and folate. Tackling these risks will likely have the added benefit of reducing enteropathogen exposure. Given the complex and multifactorial nature of cognitive development, we anticipate that the suggested interventions will likely help alleviate some of the burden of compromised cognitive development for children growing up in poverty in LMICs.
