# Supplementary material for: Early childhood cognitive development is affected by interactions among illness, diet, enteropathogens and the home environment: findings from the MAL-ED birth cohort study
Source: BMJ Glob Health. 2018 Jul 23;3(4):e000752. doi: 10.1136/bmjgh-2018-000752 (PMC6058175; doi:10.1136/bmjgh-2018-000752)
Supplement: Supplementary data [file bmjgh-2018-000752supp001.pdf]

Supplementary Materials

Supplementary Table 1: Identity and representation of variables hypothesized to be related to child development*

Domain	Variable	Representation	Included (Y/N)	Reason not in final model
SES	Crowding	Mean # of people per room in household at 24 months	N	Redundancy with the Healthy/Safe environment variable and site
	Child Cleanliness	Cleanliness Score (Factor 3 from HOME averaged across 6 and 24 months)	N	Redundancy with Health/Safe environment variable
	Environmental Safety & Healthfulness	Safety/Health Score (Factor 2 from HOME averaged across 6 and 24 months)	Y	
Intake	Breastfeeding	Proportion of days exclusively or partially breastfed from 0-6 months	N	Unrelated to the cognitive development outcomes and other variables of interest
		% days with breastmilk in diet between 6-24 months	N	Unrelated to the cognitive development outcomes and other variables of interest
		Duration of breastfeeding to 24 months	N	Unrelated to the cognitive development outcomes and other variables of interest
	Complementary Feeding	Average of monthly protein intake from meat/fish/poultry from 9-24 months	Y	
		Average of monthly vitamin B12 intake from 9-24 months	N	High correlations with vitamin B6 intake and limited number of variables that can be included in the model; because vitamin B6 had a stronger association with the outcome, it was retained in place of vitamin B12
		Average of monthly vitamin B6 intake from 9-24 months	Y	
		Average of monthly zinc intake from 9-24 months	N	Relatively lower relationship with variables of interest, some redundancy with other variables, and limited number of variables that can be included in the model
		Average of monthly folate intake from 9-24 months	Y	
		Average of monthly iron intake from 9-24 months	N	Had biomarkers of iron status that were included in the model and thought to be more closely related to cognitive development than iron intake
Micronutrient Status	Hemoglobin Concentration	Average of altitude adjusted hemoglobin concentration (7, 15, 24 months)	Y	
	Ferritin Concentration	Average of inflammation adjusted ferritin concentration (7, 15, 24 months)	N	Relatively lower relationship with variables of interest, some redundancy with other variables, and limited number of variables that can be included in the model
	Transferrin Receptor Concentration	Average of transferrin receptor concentration (7, 15, 24 months)	N	Relatively lower relationship with variables of interest, some redundancy with other variables, and limited number of variables that can be included in the model
	Zinc Concentration	Average of zinc concentration (7, 15, 24 months)	N	High rates of missing data in S. Africa site; if included, it would necessitate dropping that site
	Iodine Excretion	Average of urinary iodine excretion	N	High rates of missing data
Environmental toxin	Lead Concentration	Blood lead concentration (15 or 24 months)	N	Data missing from Brazil and Peru sites; if included, it would necessitate dropping these two sites.
Enteropathogen detection	Enteropathogens in Non-Diarrhoeal Stool	Total number of enteropathogens from non-diarrhoeal stool samples divided by number of non-diarrhoeal stools tested to 24 months	Y	
	Enteropathogens in Diarrhoeal Stool	Total number of enteropathogens from diarrhoeal stool samples	Y	

		divided by number of diarrhoeal stools tested to 24 months		
Gut Function	Lactulose Mannitol	Mean lactulose mannitol concentrations (3, 6, 9, 15 months)	N	Unrelated to the cognitive development outcomes and other variables of interest
	Fecal Myeloperoxidase	Mean concentration (monthly in year 1 and quarterly in year 2)	N	Unrelated to the cognitive development outcomes and other variables of interest
	Fecal Neopterin	Mean concentration (monthly in year 1 and quarterly in year 2)	N	Unrelated to the cognitive development outcomes and other variables of interest
	Alpha-1-Antitrypsin	Mean concentration (monthly in year 1 and quarterly in year 2)	N	Unrelated to the cognitive development outcomes and other variables of interest
Illness		Total number of days of maternal report of fever divided by days of followup	Y	
		Total number of days of maternal report of vomiting divided by days of followup	Y	
		Total number of days of ALRI divided by days of followup	Y	
		Total number of days with diarrhoeal symptoms divided by days of followup	Y	
	Alpha-1-acid glycoprotein	Systemic inflammation	N	Unrelated to the cognitive development outcomes and other variables of interest
Psychosocial Stimulation	Caregiver Responsivity	Verbal and emotional responsivity of caregiver score (Factor 1 from HOME averaged across 6 and 24 months)	N	Unrelated to the cognitive development outcomes and other variables of interest
Maternal Factors	Maternal Reasoning Ability	Total Raven's score after psychometric analyses	Y	
	Maternal Depressive Symptoms	Average of total SRQ score after psychometric analyses across 1, 6, 15 and 24 months	N[1]	SRQ scale was found to lack validity in Brazil and could not be used.
Anthropometry	Weight	Weight for age Z-score <u>in the first 17 days of life</u>	Y[2]	
Child Development	Cognitive Development	Total Bayley cognitive development score after psychometric analyses (24 months)	Y	

[*]For specifics on data collection, please see the MAL-ED Supplement in Clinical and Infectious Diseases (2014, Volume 59).

[1]Maternal depressive symptoms were omitted from the model because inclusion would have necessitated the exclusion of the BRF site, thereby, reducing the sample size and impeding our ability to conduct more complex analyses (eg, indirect effects). However, we tested this variable separately in a more basic version of the baseline model (without indirect effects) and found that it did not meaningfully influence the model.

[2]Infants were divided into tertiles by weight <u>in the first 17 days of life</u> and models were run separately for the lowest tertile and the highest tertile.

Supplementary Table 2: Model fit indices by site -- demographic variables*, configural models

	Model Fit Indices			
Model	χ^2	CFI	RMSEA	SRMR
Full Model Non-Diarrhoeal Pathogens	18·43	·99	·066	·015
Sex Invariance Model	21·65	·99	·067	·016
BGD Invariance Model	40·98	·98	·100	·025
BRF Invariance Model	14·17	·99	·048	·013
INV Invariance Model (protein intake excluded)	18·86	·99	·058	·018
PEL Invariance Model	20·15	·99	·064	·015
PKN Invariance Model	25·41	·97	·075	·017
SAV Invariance Model	15·23	·99	·051	·016

Abbreviations: Comparative Fit Index (CFI), Root Mean Square Error of Approximation (RMSEA), Standardized Root Mean Square Residual (SRMR)

*The non-invariance of the model was attributable to: (a) site differences in the relationship between B vitamin intake and illness (negative relationship at all sites except for SAV where the relationship was neutral and non-significant), and (b) site differences in the relationship between protein intake and pathogen detection (negative relationship in BRF, PKN and SAV; positive relationship in BGD and PEL; neutral and nonsignificant relationship in INV), but not to any differences in the relationship between pathogen detection and cognitive development.

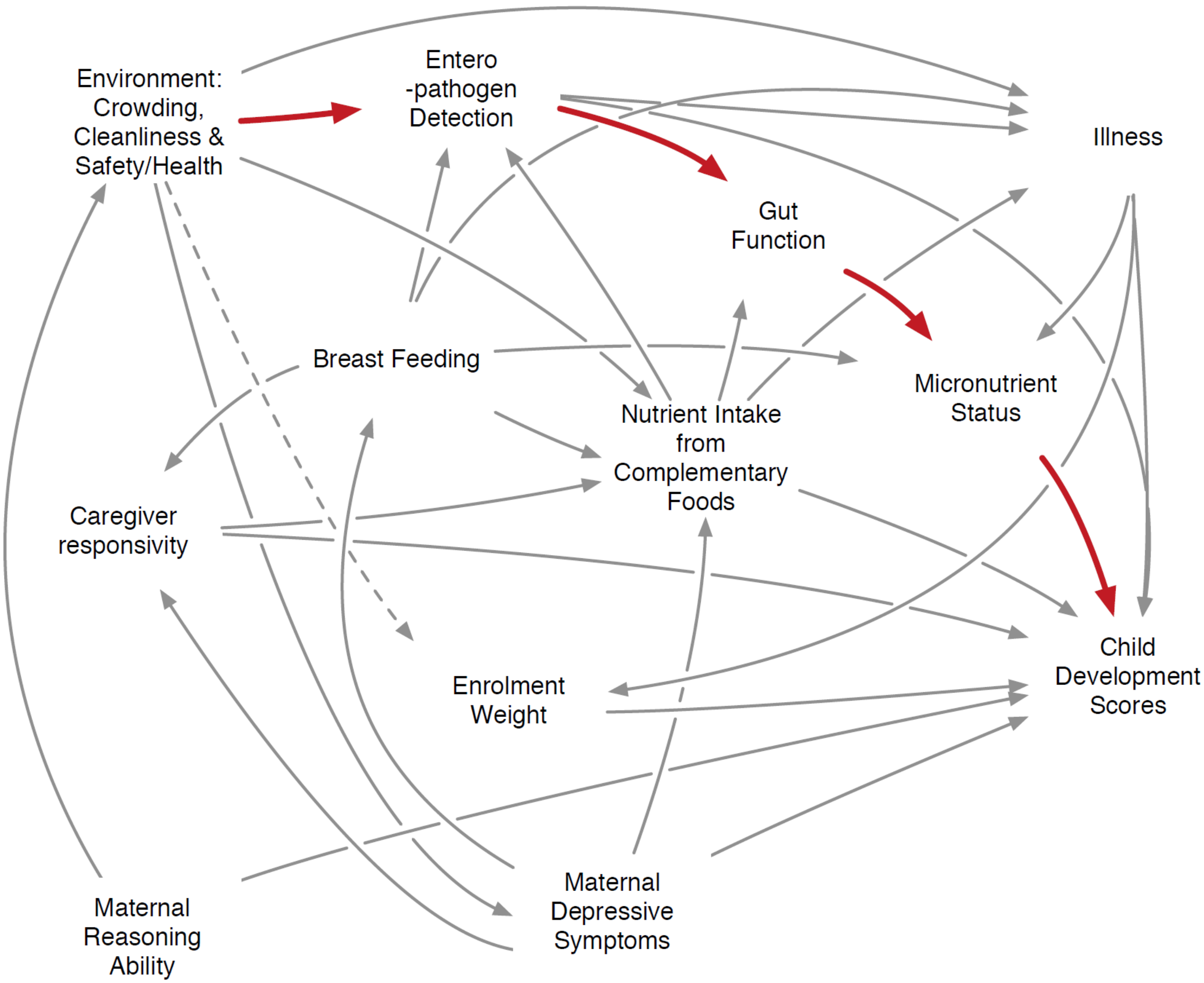

Supplementary Figure 1: Hypothesized framework for the relation between enteropathogen detection and child cognitive development (main pathway of interest indicated by maroon arrows).
